# Supplementary material for: Validation of a Phosphorus Food Frequency Questionnaire in Patients with Kidney Failure Undertaking Dialysis
Source: Nutrients. 2023 Mar 31;15(7):1711. doi: 10.3390/nu15071711 (PMC10096831; doi:10.3390/nu15071711)
Supplement: Supplementary file 1 [file nutrients-15-01711-s001.zip › nutrients-2276476-supplementary.pdf]

## Supplementary Data

## Supplemental Table S1 - Phosphate Food Frequency Questionnaire

| Food item | Never | Less than once a month | 1-2 times a month | 1-2 times a fortnight | 2-5 times a week | 1 time a day | 2 or more times a day |
|---|---|---|---|---|---|---|---|
| **Breads and cereals** | | | | | | | |
| Bread – white (including wraps, tortillas, bread rolls) | ☐ | ☐ | ☐ | ☐ | ☐ | ☐ | ☐ |
| Bread – wholemeal/ wholegrain (including wraps, tortillas, bread rolls) | ☐ | ☐ | ☐ | ☐ | ☐ | ☐ | ☐ |
| Breakfast cereal (wholegrain, e.g., bran, *Weet-bix*, muesli, rolled oats) | ☐ | ☐ | ☐ | ☐ | ☐ | ☐ | ☐ |
| Breakfast cereal (refined e.g. *Special K, Cornflakes, Rice Bubbles, Just Right*) | ☐ | ☐ | ☐ | ☐ | ☐ | ☐ | ☐ |
| Biscuits, cookies | ☐ | ☐ | ☐ | ☐ | ☐ | ☐ | ☐ |
| Cakes / scone / muffin / pancake / donuts | ☐ | ☐ | ☐ | ☐ | ☐ | ☐ | ☐ |
| **Meat, poultry, seafood, and vegetarian alternatives** | | | | | | | |
| Salami /bacon /sausages | ☐ | ☐ | ☐ | ☐ | ☐ | ☐ | ☐ |
| Processed meat – deli (e.g., ham), smoked, canned (e.g., spam) | ☐ | ☐ | ☐ | ☐ | ☐ | ☐ | ☐ |
| Meat or chicken pies / sausage rolls | ☐ | ☐ | ☐ | ☐ | ☐ | ☐ | ☐ |
| Chicken nuggets / chicken burgers | ☐ | ☐ | ☐ | ☐ | ☐ | ☐ | ☐ |
| Meat and poultry-based meals (e.g., steak, lamb, chicken, pork) | ☐ | ☐ | ☐ | ☐ | ☐ | ☐ | ☐ |
| Fresh Fish / seafood (e.g., crab, oysters) | ☐ | ☐ | ☐ | ☐ | ☐ | ☐ | ☐ |

| Food item | Never | Less than once a month | 1-2 times a month | 1-2 times a fortnight | 2-5 times a week | 1 time a day | 2 or more times a day |
|---|---|---|---|---|---|---|---|
| Pre-made or frozen seafood (e.g. battered fish, fish fingers, fish cakes, fish sticks) | □ | □ | □ | □ | □ | □ | □ |
| Canned fish (e.g., tuna, salmon, sardines) | □ | □ | □ | □ | □ | □ | □ |
| Eggs | □ | □ | □ | □ | □ | □ | □ |
| Nuts & nut spreads (e.g., peanut butter) | □ | □ | □ | □ | □ | □ | □ |
| Lentils/legumes (boiled or canned) | □ | □ | □ | □ | □ | □ | □ |
| **Dairy** | | | | | | | |
| Flavoured milk or milk shakes | □ | □ | □ | □ | □ | □ | □ |
| Milk (including soy milks) | □ | □ | □ | □ | □ | □ | □ |
| If you consume milk 2-5 times per week or more often, how much milk do you have each day? ________ cups or ___________mls *this should include milk you add to tea, coffee, breakfast cereals or drink* | | | | | | | |
| Yoghurt / Custard | □ | □ | □ | □ | □ | □ | □ |
| If you consume yoghurt / custard 2-5 times per week or more often how much yoghurt or custard do you have each day? _______grams **this should include yoghurt/custard you eat by itself, add to meals etc (1 small tub = 100g, 1 regular tub = 170g)* | | | | | | | |
| Hard cheese (e.g., cheddar, mozzarella, parmesan) | □ | □ | □ | □ | □ | □ | □ |
| Soft Cheese (e.g., cottage, ricotta, camembert) | □ | □ | □ | □ | □ | □ | □ |
| Processed Cheese (e.g., singles, cheese sticks, cheese spread) | □ | □ | □ | □ | □ | □ | □ |

| Food item | Never | Less than once a month | 1-2 times a month | 1-2 times a fortnight | 2-5 times a week | 1 time a day | 2 or more times a day |
|---|---|---|---|---|---|---|---|
| If you consume cheese (any type) 2-5 times per week or more often how much do you have each day?<br>________grams *(1 slice = 20g, 1/4c = 40g, 1 matchbox sized piece = 40g)* | | | | | | | |
| Ice-cream | ☐ | ☐ | ☐ | ☐ | ☐ | ☐ | ☐ |
| **Other** | | | | | | | |
| Puddings / instant desserts (e.g., rice pudding, crème caramel) | ☐ | ☐ | ☐ | ☐ | ☐ | ☐ | ☐ |
| Cola (e.g., Coca-Cola, Pepsi including diet or sugar free varieties) | ☐ | ☐ | ☐ | ☐ | ☐ | ☐ | ☐ |
| Beer | ☐ | ☐ | ☐ | ☐ | ☐ | ☐ | ☐ |
| Pizza | ☐ | ☐ | ☐ | ☐ | ☐ | ☐ | ☐ |
| Pasta/noodle dishes with pre-prepared/packet sauces (e.g., continental pasta, instant noodles) | ☐ | ☐ | ☐ | ☐ | ☐ | ☐ | ☐ |
| Microwave / convenience meals | ☐ | ☐ | ☐ | ☐ | ☐ | ☐ | ☐ |
| Chocolate (e.g., solid types or containing nuts) and chocolate spreads (e.g. Nutella) | ☐ | ☐ | ☐ | ☐ | ☐ | ☐ | ☐ |

Date of completion:

**Who completed this questionnaire?**

☐ Participant with no assistance

☐ Participant with some assistance

☐ Healthcare professional

Patient Identifier

## Supplemental Table S2 – 24 Hour Multi Pass Recall

**Interviewer Name ___________________________ Date of Completion:**

## 24 Hour Recall Form

*(Say to patient)* I would like for you to tell me everything you had to eat and drink all day yesterday, starting with the first thing you ate yesterday. Include everything you ate and drank at home and away – even snacks.

*(Do not interrupt patient – just fill in foods and any details they volunteer to you).*

| Meal B/L/ D/S | Food Item Brand name? | Amount Ate | Prep Method | Fat/Spices/ Condiments added | Check |
|---|---|---|---|---|---|
| | | | | | |
| | | | | | |
| | | | | | |
| | | | | | |
| | | | | | |
| | | | | | |
| | | | | | |
| | | | | | |
| | | | | | |
| | | | | | |
| | | | | | |
| | | | | | |
| | | | | | |
| | | | | | |
| | | | | | |
| | | | | | |
| | | | | | |
| | | | | | |
| | | | | | |
| | | | | | |
| | | | | | |
| | | | | | |
| | | | | | |
| | | | | | |
| | | | | | |
| | | | | | |
| | | | | | |
| | | | | | |
| | | | | | |
| | | | | | |
| | | | | | |
| | | | | | |
| | | | | | |
| | | | | | |
| | | | | | |
| | | | | | |
| | | | | | |
| | | | | | |

## 24 hr Multi Pass Recall Continued

*(After 24-hour recall is recorded, say to patient)* Now, I'm going to ask you for more detail about all the foods that you just mentioned. As we go along, when you remember anything else you ate or drank, please tell me.

*(Say to patient)* Use these measuring guides to tell me about the amounts of foods you ate. *(Show patient measuring cups and spoons, ruler, and food models).*

*Review each food with patient for the following.*

- ❑ *Record time food eaten – circle am or pm*
- ❑ *Where food was eaten – write H for home or A for away*
- ❑ *Food was for which meal or snack – write B for breakfast, L for lunch, D for dinner, S for snack*
- ❑ *Record portion size using measuring guides (see estimating portions resource)*

*Check off foods after you review them with the patient*

*(After intake details are recorded, say to the patient)* Now, let's see if I have everything.
*(Check off one box for each question)*

1) Query for item description specifically asking about

- ❑ Brand names
- ❑ How was food prepared?
- ❑ What cuts of meat were used? Is the fat trimmed? Is the fat or skin eaten?
- ❑ Are fats or condiments added? e.g. butter, margarine, mayonnaise, dressings, sauces, or gravies
- ❑ What types of fats and oils are used in cooking?
- ❑ Is salt added to cooking or at the table? How much salt is used?
- ❑ if other ingredients were added that may not be obvious

2) Did you eat or drink anything while making a meal or while waiting to eat?

- ❑ Yes, add to food list
- ❑ No

3) After you went to bed, did you wake up and have anything to eat or drink?

- ❑ Yes, add to food list
- ❑ No

4) Can you remember anything else that you ate or drank yesterday that you haven't already told me about? For example, did you have any gum, hard candy, water, tea, or ice?

- ❑ Yes, add to food list
- ❑ No

5) Lastly, was yesterday's intake what you typically consume?

- ❑ Yes
- ❑ No

  *(If no, ask)* How was yesterday different from other days? *(Record verbatim)*

*Thank patient*

**Supplemental Table S3 – Participant Demographics ESKF Cause – Other**

| Cause | Number |
|---|---|
| ESKF post CABG | 1 |
| Renal cell carcinoma | 1 |
| Mes GN type 1 | 1 |
| Glomerulonephritis | 3 |
| ESKF gradual decline | 1 |
| Primary IgA nephropathy | 1 |
| Anti – GBM anti body disease | 2 |
| Focal Segmental Glomerulosclerosis | 2 |
| NSTEMI>AKI | 1 |
| Acute tubular necrosis | 1 |
| Acquired obstructive nephropathy | 2 |
| Haemolytic Uraemia | 1 |
| Multiple myeloma | 1 |
| Ischaemic nephropathy | 1 |
